# A comprehensive study on the uric acid to high-density cholesterol ratio influencing cardiometabolic-based chronic disease in China

**DOI:** 10.3389/fnut.2026.1723098

**Published:** 2026-02-20

**Authors:** Mayina Kahaer, Yuqiu Zhu, Yi He, Wujin Chen, Dan Yang, Manxi Du, Qingqing Yang, Xiaoyu Chen, Yuping Sun, Bei Zhang

**Affiliations:** 1 Microbiology Department of Basic Medical College of Xinjiang Medical University, Xinjiang Medical University, Urumqi, China; 2 Morphological Center, Basic Medical College of Xinjiang Medical University, Urumqi, China; 3 Key Laboratory of Molecular Biology of Endemic Diseases, Xinjiang Uygur Autonomous Region, Urumqi, China; 4 Department of Basic Medicine, Xinjiang Second Medical College, Karamay, China; 5 Xinjiang Key Laboratory of Molecular Biology for Endemic Diseases, Urumqi, China

**Keywords:** cardiovascular metabolic-related chronic diseases, high-density lipoprotein cholesterol, model, nomogram, uric acid

## Abstract

Cardiovascular metabolic-related chronic diseases (CMBCD) significantly impact the quality of life and socioeconomic status of patients, yet current diagnostic and therapeutic approaches are limited by a lack of effective early screening tools and individualized treatment strategies. This study aimed to investigate the relationship between the uric acid to high-density lipoprotein cholesterol ratio (UAHDL) and CMBCD among a cohort of 3,097 participants from Xinjiang China, employing logistic regression analysis. The analysis revealed that higher UAHDL levels correlated with significant increases in age and body mass index (BMI), while a notable decline in the proportion of females and an increase in smoking and drinking prevalence were observed across UAHDL categories. Our findings identified independent risk factors for CMBCD, including female sex, older age, higher BMI, fasting blood glucose, creatinine, smoking, triglycerides, and UAHDL. Male sex was independently associated with lower odds of CMBCD. Additionally, UAHDL was significantly associated with CMBCD, and the restricted cubic spline analysis suggested a non-linear trend, with risk patterns differing across the UAHDL spectrum. Subgroup analyses further demonstrated significant associations between UAHDL and CMBCD in females and participants under 60, as well as in non-smokers and non-drinkers. We constructed a nomogram incorporating nine independent predictors, offering a practical tool for personalized risk assessment. In conclusion, our results underscore the clinical relevance of UAHDL as a potential biomarker for early identification and intervention in CMBCD. Future studies with longitudinal designs and external validation are warranted to confirm these findings and refine UAHDL-based risk stratification models.

## Introduction

Cardiovascular metabolic-related chronic diseases (CMBCD) encompass a diverse array of conditions characterized by metabolic dysregulation and increased cardiovascular risk, closely linked to cardiovascular disease (CVD)—the leading cause of global mortality (1, 2). The definition of CMBCD includes disorders such as dysglycemia, hypertension, and dyslipidemia, which may progress through distinct stages (risk development, pre-disease, disease, and complications) and ultimately lead to severe cardiovascular events like myocardial infarction or stroke (3, 4). The interplay between metabolic syndrome and cardiovascular health is particularly concerning, as factors like obesity and insulin resistance exacerbate the risk of developing heart disease (5). Consequently, understanding and addressing the underlying pathophysiology of CMBCD is critical for developing effective prevention and treatment strategies.

Current diagnostic and therapeutic approaches for CMBCD face notable limitations. Traditional methods often lack precision in early detection, fail to account for individual variability in disease progression, and overlook the dynamic staging of CMBCD (6, 7). Moreover, existing protocols are frequently generalized, neglecting personalized care—particularly in integrating early lifestyle interventions, which the CMBCD model emphasizes as pivotal for mitigating burden (8). However, current clinical practice lacks precision tools to identify individuals at risk across this full spectrum; traditional assessments often overlook early warning signs or fail to integrate the diversity of CMBCD manifestations, highlighting the need for robust biomarkers and diagnostic models.

The uric acid (UA) to high-density lipoprotein cholesterol (HDL) ratio (UAHDL) has emerged as a potential indicator, which mirrors inflammatory and metabolic conditions (9). The clinical relevance of the UAHDL ratio is rooted in the distinct biological roles played by its constituent components. UA, the end-product of purine metabolism, exhibits a dual nature. While it functions as a potent antioxidant in human plasma, scavenging oxygen, peroxyl, and hydroxyl radicals and protecting cells from oxidative damage, it can paradoxically act as a pro-oxidant, particularly within the intracellular environment (10). Elevated UA levels are implicated in the activation of the renin-angiotensin system, a reduction in insulin-induced nitric oxide synthesis in endothelial cells, and the promotion of insulin resistance (11). Conversely, high-density lipoprotein cholesterol (HDL-C) is widely recognized for its anti-inflammatory and antioxidant properties (12). HDL-C actively inhibits the oxidative modification of low-density lipoprotein (LDL) and suppresses the expression of endothelial cell adhesion molecules and monocyte chemotactic protein-1 (MCP-1), thereby exerting anti-atherogenic effects (13). From this perspective, UAHDL may better capture the balance between deleterious and protective metabolic forces than either UA or HDL-C alone.

Consistent with this biological rationale, accumulating evidence suggests that UAHDL provides superior predictive value compared with its individual components in various metabolic and cardiovascular conditions, including obesity (14), type 2 diabetes (15), metabolic syndrome (16), cardiovascular disease (17), and hypertension (18). While the relationship between UAHDL and CMBCD remains incompletely clarified, indirect findings suggest a possible link. This study aims to address this gap through a comprehensive analysis of 3,097 participants from Xinjiang, China. Using logistic regression, we investigate the relationship between UAHDL and CMBCD (encompassing hypertension, diabetes, hyperlipidemia, stroke, coronary heart disease, or Myocardial infarction). Beyond exploring this association, the study pursues three key objectives: (1) identify critical predictive factors for CMBCD; (2) develop a diagnostic model tailored to this full-spectrum definition; and (3) construct a clinically practical nomogram. By integrating these analyses, we seek to provide a robust tool for early identification of CMBCD across its entire trajectory, supporting personalized prevention and management strategies to alleviate its burden in Xinjiang, China.

## Methods

### Study population

This study retrospectively analyzed a cohort of participants. A total of 3,097 samples who participated in the health examination at the physical examination center of Barkol County Hospital in Xinjiang in 2016 were selected, including 1,210 males and 1,887 females. The ages of all the research subjects were controlled between 20 and 70 years old, and the three generations of direct blood relatives in different groups had stably resided in the sampling area for more than 20 years. This study was approved by the Ethics Committee of Xinjiang Medical University. All the research subjects gave informed consent and participated voluntarily. Participants were categorized into UAHDL tertiles (Q1–Q3) based on the sample distribution: Q1 (≤33rd percentile), Q2 (34th–66th percentile), Q3 (≥67th percentile). The exact UAHDL cut-points in the present cohort were added to Table 1 footnote.

**Table 1:** Patient demographics and baseline characteristics.

Characteristic	UAHDL group				*p*-value^2^
	Overall *N* = 3,097^1^	Q1 *N* = 1,032^1^	Q2 *N* = 1,032^1^	Q3 *N* = 1,033^1^	
Age	45 (36, 55)	43 (35, 53)	46 (36, 55)	46 (37, 57)	<0.001
BMI	24.9 (22.3, 28.0)	24.2 (21.8, 27.2)	24.7 (22.4, 27.8)	25.5 (23.0, 28.8)	<0.001
Income	2.00 (1.00, 2.00)	2.00 (1.00, 2.00)	2.00 (1.00, 2.00)	1.80 (1.00, 2.00)	0.116
SBP	131 (118, 145)	128 (115, 142)	131 (117, 145)	134 (120, 148)	<0.001
DBP	79 (70, 88)	77 (70, 86)	78 (70, 87)	80 (71, 90)	<0.001
FBG	5.17 (4.78, 5.58)	5.10 (4.73, 5.51)	5.18 (4.82, 5.58)	5.23 (4.81, 5.66)	<0.001
ALT	17 (13, 22)	15 (12, 20)	16 (13, 21)	20 (15, 27)	<0.001
AST	17.0 (14.0, 20.0)	16.0 (14.0, 19.0)	16.0 (14.0, 19.0)	17.0 (15.0, 21.0)	<0.001
Gln	24 (16, 39)	18 (13, 28)	23 (17, 37)	32 (22, 52)	<0.001
ALP	90 (73, 110)	80 (65, 99)	89 (73, 108)	101 (85, 120)	<0.001
BUN	4.60 (4.00, 5.50)	4.30 (3.70, 5.10)	4.60 (4.00, 5.40)	5.00 (4.30, 5.70)	<0.001
CRE	58 (51, 68)	52 (48, 58)	58 (51, 67)	67 (59, 74)	<0.001
UA	253 (209, 305)	195 (171, 222)	254 (229, 280)	322 (288, 361)	<0.001
TG	1.03 (0.76, 1.51)	0.81 (0.63, 1.07)	1.02 (0.79, 1.39)	1.40 (1.02, 2.05)	<0.001
TC	5.16 (4.43, 5.90)	5.07 (4.46, 5.77)	5.14 (4.38, 5.86)	5.25 (4.43, 6.04)	0.005
HDL	1.37 (1.16, 1.60)	1.64 (1.48, 1.85)	1.38 (1.24, 1.53)	1.11 (0.99, 1.24)	<0.001
LDL	3.01 (2.48, 3.59)	2.71 (2.30, 3.23)	3.02 (2.51, 3.58)	3.31 (2.74, 3.91)	<0.001
UAHDL	3.11 (2.32, 4.12)	2.05 (1.75, 2.32)	3.11 (2.83, 3.38)	4.71 (4.12, 5.59)	<0.001
Sex					<0.001
Female	1,887 (60.9%)	876 (84.9%)	648 (62.8%)	363 (35.1%)	
Male	1,210 (39.1%)	156 (15.1%)	384 (37.2%)	670 (64.9%)	
Education					0.058
Graduate	196 (6.3%)	52 (5.0%)	61 (5.9%)	83 (8.0%)	
High	836 (27.0%)	284 (27.5%)	289 (28.0%)	263 (25.5%)	
Junior	1,843 (59.5%)	632 (61.2%)	606 (58.7%)	605 (58.6%)	
Primary	222 (7.2%)	64 (6.2%)	76 (7.4%)	82 (7.9%)	
Smoking					<0.001
No	2,546 (82.2%)	970 (94.0%)	856 (82.9%)	720 (69.7%)	
Yes	551 (17.8%)	62 (6.0%)	176 (17.1%)	313 (30.3%)	
Drinking					<0.001
No	2,912 (94.0%)	1,019 (98.7%)	978 (94.8%)	915 (88.6%)	
Yes	185 (6.0%)	13 (1.3%)	54 (5.2%)	118 (11.4%)	
Hypertension					0.025
No	2,155 (69.6%)	750 (72.7%)	709 (68.7%)	696 (67.4%)	
Yes	942 (30.4%)	282 (27.3%)	323 (31.3%)	337 (32.6%)	
Diabetes					0.134
No	3,014 (97.3%)	1,007 (97.6%)	1,010 (97.9%)	997 (96.5%)	
Yes	83 (2.7%)	25 (2.4%)	22 (2.1%)	36 (3.5%)	
Hyperlipidemia					0.206
No	3,049 (98.5%)	1,021 (98.9%)	1,016 (98.4%)	1,012 (98.0%)	
Yes	48 (1.5%)	11 (1.1%)	16 (1.6%)	21 (2.0%)	
Stroke					0.519
No	3,077 (99.4%)	1,027 (99.5%)	1,023 (99.1%)	1,027 (99.4%)	
Yes	20 (0.6%)	5 (0.5%)	9 (0.9%)	6 (0.6%)	
CHD					0.157
No	2,718 (87.8%)	922 (89.3%)	900 (87.2%)	896 (86.7%)	
Yes	379 (12.2%)	110 (10.7%)	132 (12.8%)	137 (13.3%)	
MI					0.846
No	3,079 (99.4%)	1,027 (99.5%)	1,025 (99.3%)	1,027 (99.4%)	
Yes	18 (0.6%)	5 (0.5%)	7 (0.7%)	6 (0.6%)	
CMBCD					0.013
No	1,921 (62.0%)	677 (65.6%)	627 (60.8%)	617 (59.7%)	
Yes	1,176 (38.0%)	355 (34.4%)	405 (39.2%)	416 (40.3%)	

^1^Median (Q1, Q3); *n* (%).

^2^Kruskal-Wallis rank sum test; Pearson’s Chi-squared test. BMI, body mass index (kg/m*^2^*); SBP, systolic blood pressure (mmHg); DBP, diastolic blood pressure (mmHg); FBG, fasting blood glucose (mmol/L); ALT, alanine aminotransferase (U/L); AST, aspartate aminotransferase (U/L); Gln (γ-glutamyl transferase, U/L); ALP, alkaline phosphatase (U/L); BUN, blood urea nitrogen (mmol/L); CRE, creatinine (μmol/L); UA, uric acid (μmol/L); TG, triglycerides (mmol/L); TC, total cholesterol (mmol/L); HDL, high-density lipoprotein cholesterol (mmol/L); LDL, low-density lipoprotein cholesterol (mmol/L); UAHDL, uric acid / HDL-C ratio; CHD, coronary heart disease; MI, myocardial infarction; CMBCD, cardiometabolic-based chronic disease. Participants were categorized into UAHDL tertiles (Q1–Q3) based on the sample distribution: Q1 (≤33rd percentile), Q2 (34th–66th percentile), Q3 (≥67th percentile).

### Data collection and variable definitions

Comprehensive data were collected for each participant, including demographic information (e.g., sex, age), anthropometric measurements (e.g., body mass index [BMI]), clinical and hemodynamic measurements (Systolic Blood Pressure [SBP], Diastolic Blood Pressure [DBP]), lifestyle factors (e.g., smoking status), and biochemical parameters from blood and urine samples (e.g., UAHDL, Fasting Blood Glucose [FBG], creatinine [CRE], Triglycerides [TG], total cholesterol [TC], blood urea nitrogen [BUN], low-density lipoprotein cholesterol [LDL]). All laboratory measurements were performed using standardized protocols in a certified clinical laboratory. Cardiometabolic-based chronic disease (CMBCD) was defined as the presence of at least one of the coronary heart disease, myocardial infarction, stroke, diabetes, hypertension, and dyslipidemia in a single individual. For ascertainment: (1) CHD, MI and stroke were identified from the participant’s medical record and confirmed by discharge diagnosis or imaging/ECG documentation where available; (2) diabetes was defined as fasting blood glucose (FBG) ≥ 7.0 mmol/L, self-reported physician diagnosis of diabetes, or current use of glucose-lowering medication; (3) hypertension was defined as systolic blood pressure ≥ 140 mmHg or diastolic blood pressure ≥ 90 mmHg, self-reported physician diagnosis, or current antihypertensive medication; (4) dyslipidemia was defined as total cholesterol, LDL-C, HDL-C, or TG outside laboratory reference ranges, self-reported diagnosis, or current lipid-lowering therapy.

### Descriptive statistics for baseline characteristics

The analysis included measures such as mean (standard deviation) for normally distributed variables and median (IQR) for non-normally distributed variables for continuous variables, and frequencies and percentages for categorical variables. Normality tests and QQ plots were used to assess the distribution of the data, and appropriate descriptive statistics methods were applied to both normally and non-normally distributed variables. Group comparisons for continuous variables with normal distribution were performed using Welch’s t-test or ANOVA, and non-normally distributed variables were compared using the Wilcoxon rank-sum test or Kruskal-Wallis test. For comparison between groups of categorical data, we used the Fisher exact test for expected frequencies <5; otherwise, we used the Chi-squared test.

In our study, all statistical analyses were performed using the R software (version 4.2.2), along with MSTATA software.^1^

### A comprehensive investigation of independent clinical factors influencing CMBCD

Univariate logistic regression was performed for a broad set of candidate predictors—including demographic, anthropometric, hemodynamic and laboratory variables (sex, age, BMI, education, smoking, drinking, SBP, DBP, FBG, ALT, AST, Gln, ALP, BUN, CRE, UA, TG, TC, HDL, LDL, UAHDL etc.). Variables with *p* < 0.10 in univariate analysis or judged clinically important were considered for multivariable modeling. Multivariable models were constructed using clinical knowledge and stepwise selection. This comprehensive approach ensured that all potential influencing factors were considered and adjusted for, providing a more accurate estimation of the independent clinical factors that significantly impacted CMBCD.

Subgroup analyses were conducted based on age, sex, BMI, education, smoking, and drinking to explore potential effect modifiers and assess the robustness of the main findings across different patient populations.

### Investigating the relationship between UAHDL and CMBCD using restricted cubic splines

Possible nonlinear relationships between the change in UAHDL and CMBCD were examined by a Logistic regression model with RCS. The knots between 3 and 7 were tested respectively, and the model with lowest Akaike information criterion value was selected for RCS. Finally, we used RCS with 5 knots at the 5th, 28th, 50th, 72th, and 95th percentiles.

To assess the overall association between UAHDL and CMBCD, we examined the global *p*-overall. *p*-overall < 0.05 indicates a statistically significant overall association between UAHDL and CMBCD, suggesting that the relationship modeled by the RCS differs significantly from a null hypothesis of no association. Additionally, we assessed whether the association between UAHDL and CMBCD is nonlinear. We considered *p*-nonlinear <0.05 as evidence for statistically significant nonlinearity; however, results with *p*-nonlinear close to 0.05 were interpreted cautiously.

### Nomogram construction and validation

Based on the significant independent variables (*p* < 0.05) identified in the multivariable logistic regression analysis, a nomogram was constructed to predict the probability of CMBCD. Calibration curves were generated to assess the agreement between the predicted probabilities from the nomogram and the observed CMBCD rates. Both apparent and bootstrap bias-corrected calibration curves were plotted. Internal validation was performed using a bootstrap resampling procedure with 1,000 repetitions to obtain optimism-corrected estimates of model performance. The calibration plot was visually inspected, and the consistency between the apparent and bias-corrected curves was evaluated against the ideal reference line. Model calibration was further quantified using the calibration intercept and slope, as well as the maximum and average calibration errors (Emax and Eavg). Discriminative ability was evaluated by calculating the concordance index (C-statistic), and overall predictive accuracy was assessed using the Brier score. The clinical utility of the nomogram was further assessed using decision curve analysis (DCA). DCA was performed to quantify the net benefit of using the nomogram across a range of threshold probabilities for intervention, comparing it against the strategies of intervening on all or no patients. A two-tailed *p*-value of less than 0.05 was considered statistically significant for all analyses.

## Results

### Descriptive statistics for baseline characteristics

Table 1 presents the baseline characteristics of 3,097 participants stratified by UAHDL groups (Q1–Q3). Significant differences were observed across groups for most continuous variables, including age (median [IQR]: Q1 = 43 [35, 53], Q2 = 46 [36, 55], Q3 = 46 [37, 57], *p* < 0.001), BMI (Q1 = 24.2 [21.8, 27.2], Q2 = 24.7 [22.4, 27.8], Q3 = 25.5 [23.0, 28.8], *p* < 0.001), and cardiometabolic markers such as SBP, DBP, FBG, and lipid profiles (all *p* < 0.001). Notable differences were found in liver enzymes (ALT: Q1 = 15 [12, 20], Q3 = 20 [15, 27]; AST: Q1 = 16 [14, 19], Q3 = 17 [15, 21]) and renal function markers (CRE: Q1 = 52 [48, 58], Q3 = 67 [59, 74]; BUN: Q1 = 4.3 [3.7, 5.1], Q3 = 5.0 [4.3, 5.7]). Categorical variables revealed significant sex distribution differences (female: Q1 = 84.9%, Q3 = 35.1%), with higher smoking (Q1 = 6.0%, Q3 = 30.3%) and drinking prevalence (Q1 = 1.3%, Q3 = 11.4%) in Q3. Hypertension prevalence increased across groups (Q1 = 27.3%, Q3 = 32.6%, *p* = 0.025), while diabetes, hyperlipidemia, stroke, CHD, MI and hyperlipidaemia showed no significant differences. The CMBCD showed higher prevalence in Q3 (40.3% vs. Q1 34.4%, *p* = 0.013).

### A comprehensive investigation of independent clinical factors influencing CMBCD

**Figure 1:**
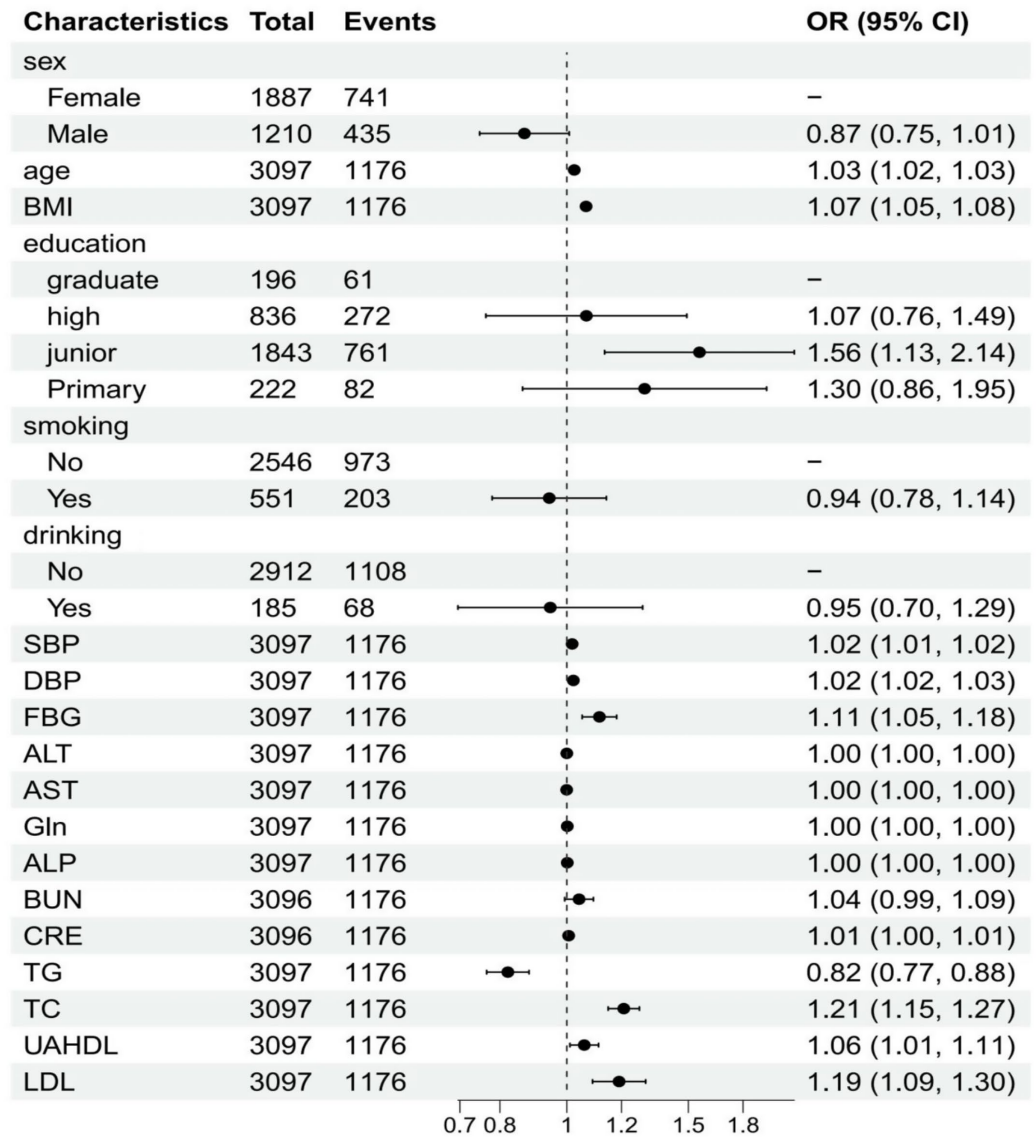
Univariate analysis of influencing factors (logistic regression).

In the univariate analysis, several factors demonstrated significant associations with CMBCD. Male sex showed a protective effect with an odds ratio (OR) of 0.87 (95% CI: 0.75, 1.01), though its *p*-value (0.063) was borderline significant. Age was positively associated with CMBCD (OR = 1.03, 95% CI: 1.02, 1.03, *p* < 0.001), as was BMI (OR = 1.07, 95% CI: 1.05, 1.08, *p* < 0.001). UAHDL (OR = 1.06, 95% CI: 1.01, 1.11, *p* = 0.017), systolic blood pressure (SBP) (OR = 1.02, 95% CI: 1.01, 1.02, *p* < 0.001), and fasting blood glucose (FBG) (OR = 1.11, 95% CI: 1.05, 1.18, *p* < 0.001) were also significantly associated with increased odds of CMBCD. Conversely, triglycerides (TG) (OR = 0.82, 95% CI: 0.77, 0.88, *p* < 0.001) and total cholesterol (TC) (OR = 1.21, 95% CI: 1.15, 1.27, *p* < 0.001) showed opposing associations. Regarding education, individuals with a junior level had significantly higher odds of CMBCD (OR = 1.56, 95% CI: 1.13, 2.14, *p* = 0.006) compared to graduates. Among laboratory parameters, creatinine (CRE) (OR = 1.01, 95% CI: 1.00, 1.01, *p* = 0.014) and LDL (OR = 1.19, 95% CI: 1.09, 1.30, *p* < 0.001) also showed significant associations (Figure 1).

**Figure 2:**
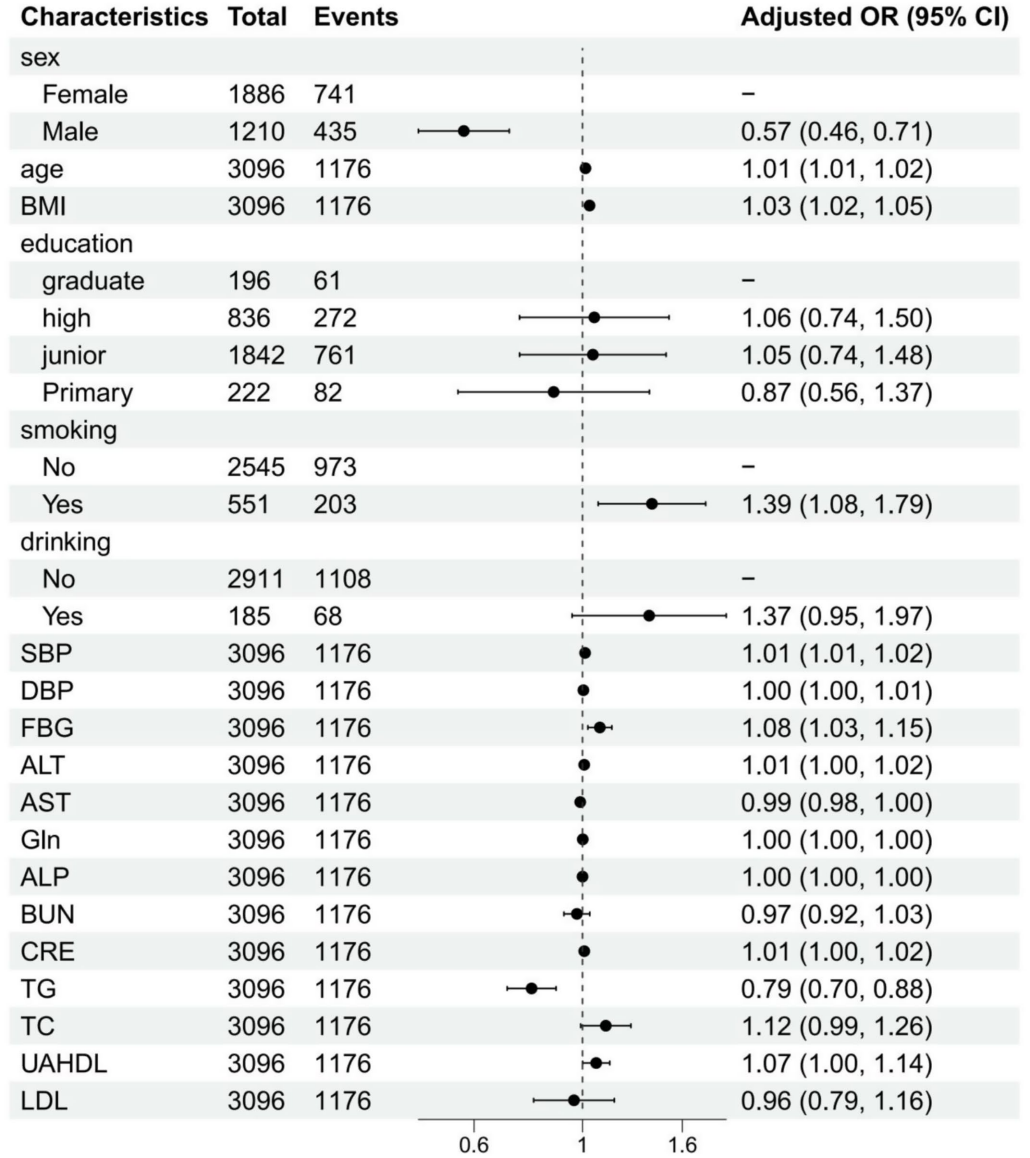
Multivariate analysis of influencing factors (logistic regression).

After adjusting for potential confounders in the multivariable regression analysis, several independent associations remained significant. Male sex emerged as a protective factor against CMBCD (OR = 0.57, 95% CI: 0.46, 0.71, *p* < 0.001) when compared to females. Age (OR = 1.01, 95% CI: 1.01, 1.02, *p* < 0.001) and BMI (OR = 1.03, 95% CI: 1.02, 1.05, *p* < 0.001) continued to show significant positive associations. UAHDL also remained significantly associated with CMBCD (OR = 1.07, 95% CI: 1.00, 1.14, *p* = 0.048). FBG maintained its strong positive association (OR = 1.08, 95% CI: 1.03, 1.15, *p* = 0.005), as did CRE (OR = 1.01, 95% CI: 1.00, 1.02, *p* = 0.024). Smoking status became a significant risk factor in the multivariable model, with current smokers having increased odds of CMBCD (OR = 1.39, 95% CI: 1.08, 1.79, *p* = 0.011). Notably, TG continued to show a protective effect (OR = 0.79, 95% CI: 0.70, 0.88, *p* < 0.001). Conversely, the previously significant association of junior education and LDL diminished in the multivariable model, indicating their effects might have been confounded by other variables (Figure 2).

### Investigating the relationship between UAHDL and CMBCD using restricted cubic splines

**Figure 3:**
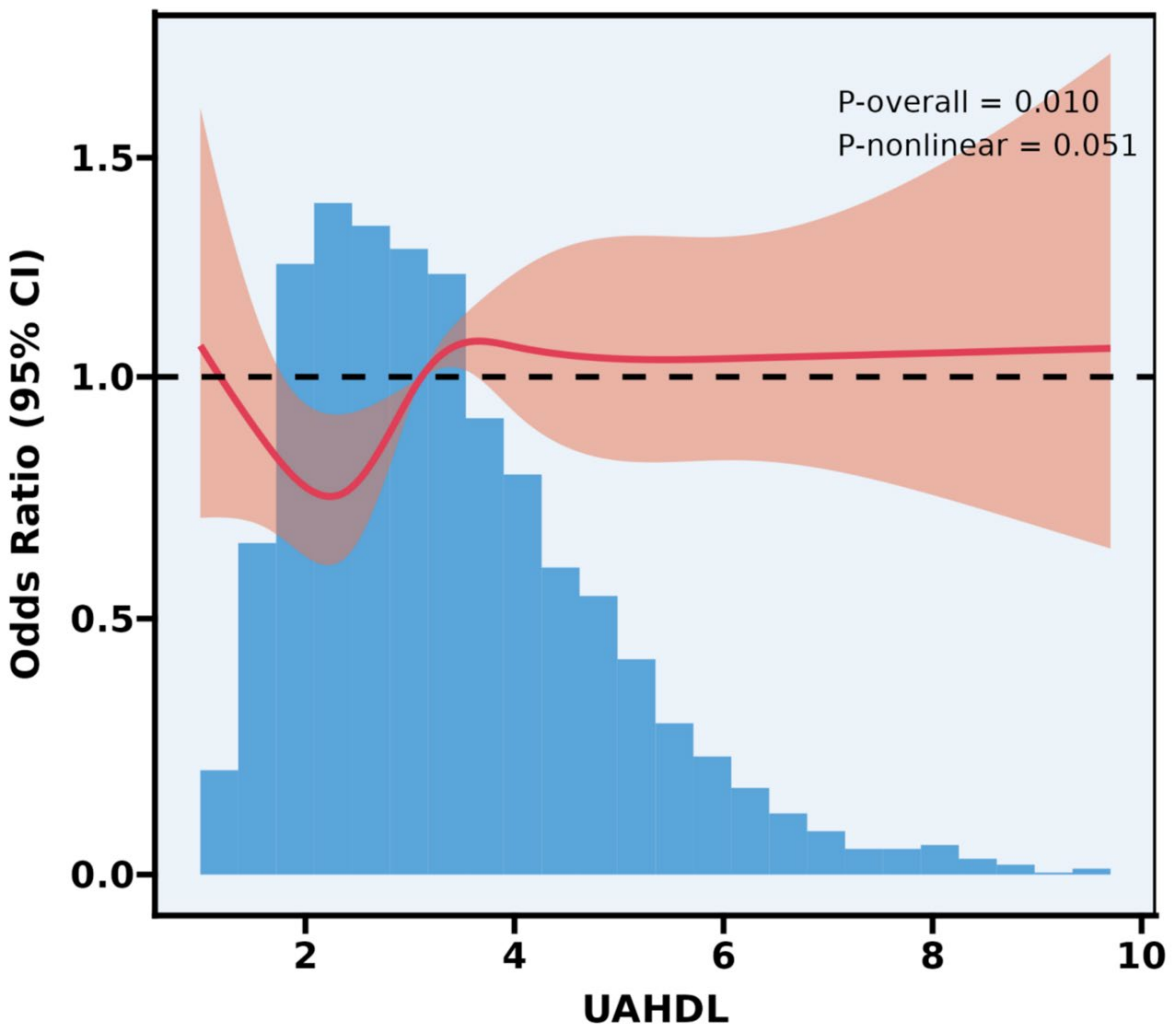
Association between UAHDL and CMBCD with the RCS function. Model with 5 knots located at 5th, 28th, 50th, 72th, and 95th percentiles. Y-axis represents the OR to present CMBCD for any value of UAHDL compared to individuals with reference value (50th percentile) of UAHDL.

Restricted cubic splines were employed to investigate the complex relationship between UAHDL and the odds of CMBCD in the target population (Figure 3). *p*-overall = 0.010 indicates a significant overall association; *p*-nonlinear = 0.051 is borderline, suggesting a suggestive (but not definitively statistically significant) nonlinearity (approximately U-shaped). Specifically, lower UAHDL values (approximately below 2.5–3.0) were associated with a decreasing odds ratio for CMBCD, indicating a potential protective effect, with the lowest odds observed around UAHDL values of 2.0–2.5. Beyond this nadir, the odds ratio for CMBCD gradually increased, approaching and then slightly exceeding 1.0 for higher UAHDL concentrations, although remaining relatively stable across the majority of the population distribution (UAHDL values between approximately 3.0 and 6.0). This suggests that while very low UAHDL may be associated with increased risk, the benefit appears to diminish, and potentially reverse, at higher concentrations, highlighting a nuanced dose–response pattern.

### Subgroup analysis

Subgroup analysis were conducted to investigate the association between UAHDL and CMBCD across various baseline characteristics. In the univariate analysis (Table 2), significant associations were consistently observed in females (OR 1.17, 95% CI 1.09–1.27, *p* < 0.001) and in patients aged <60 years (OR 1.06, 95% CI 1.01–1.12, *p* = 0.027). Furthermore, significant associations were found in non-smokers (OR 1.07, 95% CI 1.01–1.13, *p* = 0.019) and non-drinkers (OR 1.07, 95% CI 1.02–1.12, *p* = 0.008). The interaction term for sex was also significant (*p* = 0.017). Upon adjustment for covariates, the significant association in females remained (OR 1.16, 95% CI 1.05–1.27, *p* = 0.002), as did the association in patients aged <60 years (OR 1.09, 95% CI 1.02–1.17, *p* = 0.014). A significant association was observed in participants with BMI < 24 kg/m^2^ (OR 1.11, 95% CI 1.00–1.23, *p* = 0.042), whereas no significant associations were found in those with BMI 24–27.9 kg/m^2^ or ≥28 kg/m^2^. In addition, the significant association in participants with high school education became more pronounced after adjustment (OR 1.18, 95% CI 1.04–1.34, *p* = 0.011). The significant association in non-drinkers was sustained (OR 1.07, 95% CI 1.00–1.15, *p* = 0.036), while the association in non-smokers was attenuated and no longer statistically significant after adjustment (OR 1.07, 95% CI 0.99–1.15, *p* = 0.070). The interaction with sex also remained significant in the adjusted model (*p* for interaction = 0.010).

**Table 2:** Subgroup analysis.

Variables	*n* (%)	OR (95%CI)^a^	*p* ^a^	*p* for interaction^a^	OR (95%CI)^b^	*p* ^b^	*p* for interaction^b^
Sex				0.017			0.010
Female	1,887 (60.93)	1.17 (1.09–1.27)	<0.001		1.16 (1.05–1.27)	0.002	
Male	1,210 (39.07)	1.03 (0.96–1.11)	0.403		1.00 (0.91–1.09)	0.967	
Age				0.436			0.178
<60	2,563 (82.76)	1.06 (1.01–1.12)	0.027		1.09 (1.02–1.17)	0.014	
≥60	534 (17.24)	1.01 (0.91–1.13)	0.826		0.96 (0.83–1.11)	0.570	
BMI				0.121			0.248
BMI < 24	54 (1.74)	1.00 (0.65–1.52)	0.994		1.11 (1.00–1.23)	0.042	
24 ≤ BMI < 28	1,534 (49.53)	1.05 (0.97–1.13)	0.243		0.92 (0.81–1.04)	0.166	
BMI ≥ 28	448 (14.47)	1.06 (0.95–1.18)	0.286		1.12 (0.99–1.27)	0.062	
Education				0.433			0.342
Graduate	196 (6.33)	0.99 (0.81–1.23)	0.960		0.89 (0.62–1.28)	0.536	
High	836 (26.99)	1.10 (1.00–1.20)	0.057		1.18 (1.04–1.34)	0.011	
Junior	1,843 (59.51)	1.04 (0.98–1.11)	0.222		1.04 (0.96–1.13)	0.372	
Primary	222 (7.17)	1.19 (0.99–1.42)	0.064		1.06 (0.83–1.37)	0.624	
Smoking				0.936			0.831
No	2,546 (82.21)	1.07 (1.01–1.13)	0.019		1.07 (0.99–1.15)	0.070	
Yes	551 (17.79)	1.06 (0.95–1.19)	0.288		1.09 (0.95–1.25)	0.234	
Drinking				0.286			0.258
No	2,912 (94.03)	1.07 (1.02–1.12)	0.008		1.07 (1.00–1.15)	0.036	
Yes	185 (5.97)	0.95 (0.76–1.18)	0.628		1.07 (0.81–1.43)	0.631	

OR, Odds Ratio; CI, Confidence Interval. ^a^Crude analysis. ^b^Adjusted by Sex, Age, BMI, Education, Smoking, drinking, SBP, DBP, FBG, ALT, AST, Gln, ALP, BUN, CRE, UA, TG, TC, HDL, LDL.

### Nomogram development

**Figure 4:**
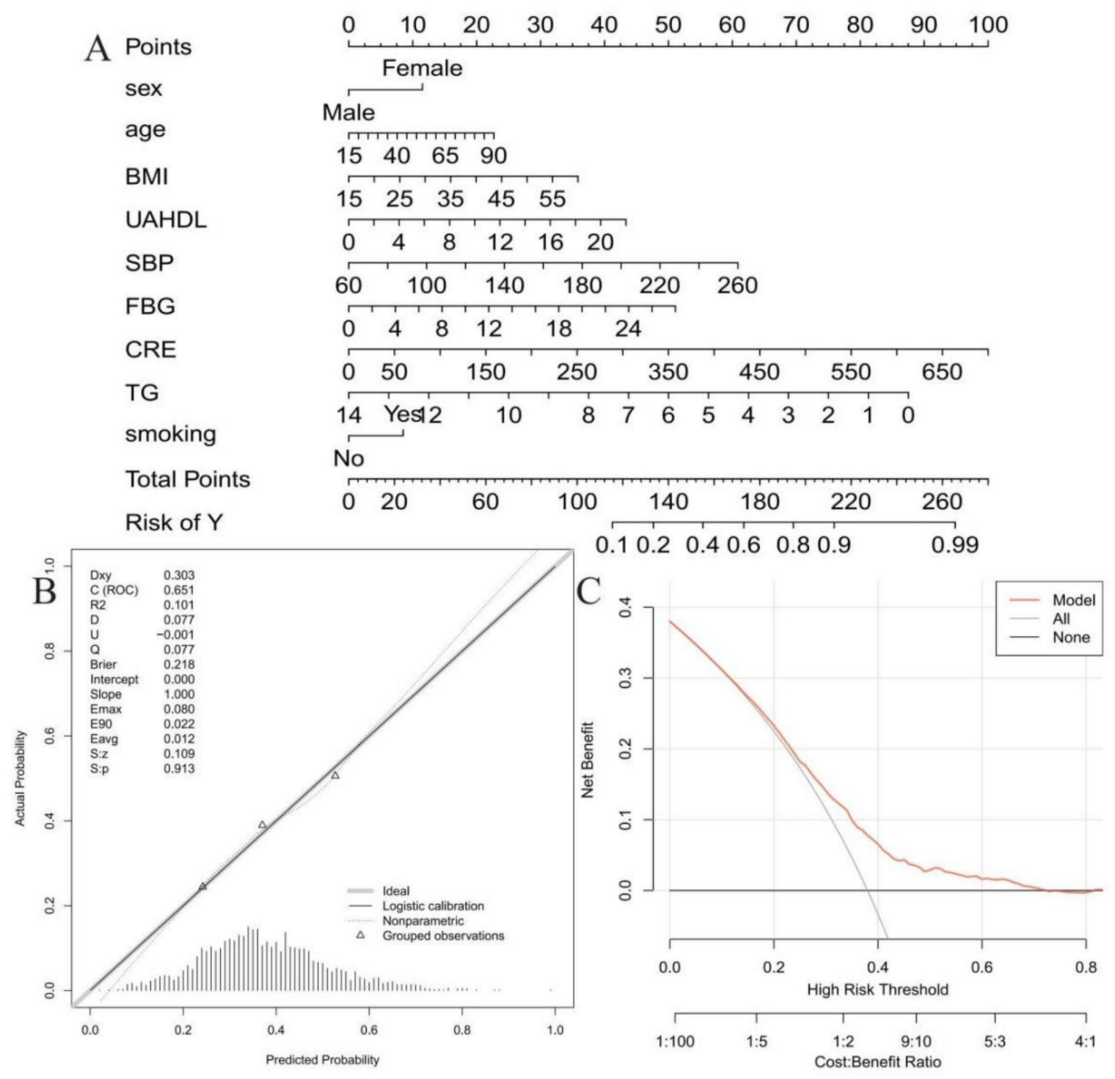
Nomogram for predicting CMBCD risk and its validation. **(A)** Nomogram for predicting individual risk of CMBCD. **(B)** Calibration curve of the nomogram. **(C)** Decision curve analysis (DCA) of the nomogram.

Building upon the multivariable analysis, nine variables with a *p*-value less than 0.05 were identified as independent predictors of CMBCD: sex, age, BMI, UAHDL, SBP, FBG, CRE, TG, and smoking. These variables were subsequently used to construct a nomogram (Figure 4A). This nomogram serves as a practical, visual tool for individualized risk prediction. To utilize the nomogram, points are assigned to each variable based on its specific value or category, summed to obtain a “Total Points” score, which then correlates directly to the “Risk of Y” (the predicted probability of CMBCD).

Then, the predictive performance and clinical utility of the nomogram were rigorously evaluated through calibration curve analysis and decision curve analysis (DCA). As shown in Figure 4B, the calibration plot demonstrated good agreement between the predicted and observed probabilities, with the apparent and bias-corrected curves closely approximating the ideal reference line. The model exhibited a C-statistic of 0.651 and a Brier score of 0.218, indicating acceptable discrimination and overall predictive accuracy. Moreover, the calibration intercept was 0.000 and the slope was 1.000, suggesting no evident systematic over- or underestimation of risk. The low calibration error (Emax = 0.080, Eavg = 0.012) further supported the good calibration performance of the nomogram. The decision curve analysis (Figure 4C) further confirmed the nomogram’s clinical utility. The corrected C-statistic of the nomogram model obtained from bootstrap resampling was 0.646, indicating good internal validation. Over a broad range of high-risk thresholds (approximately 0.05–0.7), the nomogram consistently yielded a higher net benefit compared to strategies of treating all patients or no patients. This suggests that incorporating the nomogram into clinical decision-making would lead to improved outcomes by optimizing intervention strategies and balancing the benefits of true positives against the harms of false positives.

## Discussion

This study comprehensively investigated the uric acid to high-density lipoprotein cholesterol ratio (UAHDL) and cardiometabolic-based chronic diseases (CMBCD) in a 3,097-participant cohort from Xinjiang, China. We identified UAHDL as an independent predictor of CMBCD and observed an approximately U-shaped, non-linear association, wherein both very low and very high ratios increased risk. Other independent predictors included male gender, age, BMI, FBG, CRE, smoking, and TG. A validated nomogram was developed using these nine factors for personalized risk assessment. These findings are crucial for early CMBCD screening and individualized treatment, addressing current limitations in precision tools.

The independent association of UAHDL with CMBCD, as observed in this study, aligns with previous research that has linked this ratio to various metabolic conditions, including obesity, type 2 diabetes, metabolic syndrome, and cardiovascular diseases (19, 20). Baseline analyses further demonstrated that higher UAHDL levels clustered with advanced age, higher BMI, and a greater prevalence of smoking and drinking behaviors, all of which are well-established correlates of metabolic and cardiometabolic dysfunction. These patterns support UAHDL as an integrative biomarker capturing cumulative metabolic burden rather than an isolated biochemical abnormality.

Given these opposing functions, the UAHDL ratio serves as a comprehensive index reflecting the balance between pro-oxidant/pro-inflammatory (21) and antioxidant/anti-inflammatory (HDL-C) (22) processes. An elevated UAHDL ratio therefore indicates a shift toward systemic oxidative stress and inflammation, which are fundamental pathogenic mechanisms in the development and progression of CMBCD. The synergistic effect, where the combination of high UA and low HDL-C leads to greater endothelial dysfunction and oxidative stress than either factor alone, provides a strong mechanistic foundation for the ratio’s predictive value in cardiometabolic health (23). Importantly, the increased risk observed at very low UAHDL levels may reflect reduced antioxidant capacity due to abnormally low UA or impaired HDL functionality rather than a protective metabolic state. UA is a major circulating antioxidant, and excessively low levels have been associated with increased oxidative stress and adverse cardiovascular outcomes (24, 25). In parallel, accumulating evidence suggests that HDL quantity does not necessarily equate to functionality, as dysfunctional HDL may lose its protective effects under inflammatory conditions (26). Together, these mechanisms provide a plausible explanation for the observed U-shaped association, suggesting that both extremes of the UAHDL spectrum may confer cardiometabolic risk. Rather than a simple linear relationship, these findings support the concept of an optimal physiological range of UAHDL, within which metabolic homeostasis is maintained.

Multivariable analysis identified age, BMI, FBG, CRE, and smoking as independent predictors in metabolic syndrome and cardiovascular disease globally, consistent with their established roles (27, 28). Male sex emerged as a protective factor (OR = 0.57, *p* < 0.001), which diverges from general epidemiological trends where women often face greater mortality risk from cardiometabolic dysfunction (29). This indicated that male sex was associated with a lower risk of CMBCD, a finding that likely reflects sex-specific susceptibility to metabolic disturbances rather than a lower baseline risk in men. Extensive evidence has demonstrated that the relative risk of cardiovascular outcomes conferred by metabolic conditions, particularly diabetes, is significantly higher in women than in men (30). This exacerbated impact of risk factors in the female population potentially leads to a higher disease burden compared to males with similar metabolic profiles, thus resulting in the observed lower adjusted odds for men in our cohort. Subgroup analyses showed significant UAHDL associations in females (adjusted OR 1.15, *p* = 0.004), those under 60 years (adjusted OR 1.09, *p* = 0.021), non-smokers, and non-drinkers (31). The persistent association in females and the significant sex interaction term (*p* = 0.008) highlight sex-specific vulnerabilities (32). The association in younger individuals suggests UAHDL’s importance as an early risk indicator (33).

Intriguingly, while TC maintained its role as a robust risk factor, TG exhibited an inverse association, mirroring the ‘triglyceride paradox’ often reported in cardiovascular research. This divergence is likely driven by nutritional status and metabolic reserve rather than a direct protective mechanism of lipids. Low TG levels may serve as surrogates for malnutrition or frailty, akin to the ‘obesity paradox’ where adequate energy stores confer survival advantages (34). Furthermore, consistent with the U-shaped mortality risk observed with the triglyceride-glucose index, physiological substrates falling below a critical threshold often indicate catabolic states and predict poorer prognosis (35). Biologically, extremely low TG might compromise cell membrane stability and reduce buffering against fatty acid-induced lipotoxicity (34), suggesting that the observed ‘protective’ link in our study reflects preserved energy homeostasis.

Metabolic syndrome represents a core cluster of cardiometabolic abnormalities characterized by central obesity, insulin resistance, hypertension, and dyslipidemia, all of which substantially overlap with the CMBCD framework. Emerging evidence has demonstrated that UAHDL is strongly associated with MetS prevalence and severity across diverse populations, often outperforming UA or HDL-C alone as a risk indicator (36). Elevated UAHDL has been linked to increased insulin resistance, systemic inflammation, and endothelial dysfunction, key mechanisms underlying MetS development (37). Although MetS was not evaluated as a distinct outcome in the present analysis, the strong association between UAHDL and CMBCD observed in our study likely reflects shared metabolic pathways with MetS. Future studies incorporating standardized MetS definitions (e.g., ATP III or IDF criteria) and longitudinal designs are warranted to further clarify the role of UAHDL in MetS risk stratification and progression.

The nomogram, incorporating nine independent predictors, is a significant step toward personalized CMBCD risk assessment, addressing the need for precision tools. Its robust predictive performance, validated by calibration curve analysis and DCA, indicates reliability and clinical utility (38). This tool can optimize intervention strategies by balancing benefits and harms, facilitating earlier, more informed clinical decisions.

Several limitations merit consideration. First, the cross-sectional design precludes causal inference, and temporal relationships between UAHDL and CMBCD cannot be established. Second, although diagnostic criteria for each CMBCD component were clarified, the composite outcome inherently overlaps with several predictors (e.g., SBP, FBG, lipid parameters), raising the possibility of incorporation bias and inflation of predictive performance. Third, high-sensitivity C-reactive protein and leukocyte differentials were unavailable, preventing evaluation of inflammatory indices such as CRP, SIRI, or SII, which may further elucidate the inflammatory relevance of UAHDL. Fourth, this model underwent internal validation only; external validation in independent and ethnically diverse populations is required before clinical implementation. Finally, residual confounding from unmeasured lifestyle and genetic factors cannot be excluded.

In conclusion, UAHDL is independently associated with CMBCD and exhibits a complex risk pattern across its distribution. Combined with established metabolic risk factors, UAHDL contributes meaningfully to CMBCD risk stratification. The proposed nomogram provides a clinically accessible framework for individualized assessment, although prospective validation is essential before routine application.

## Data Availability

The original contributions presented in the study are included in the article/supplementary material, further inquiries can be directed to the corresponding authors.

## Footnotes

1
www.mstata.com

## References

[1] Mechanick JI Farkouh ME Newman JD Garvey WT. Cardiometabolic-based chronic disease, adiposity and dysglycemia drivers: JACC state-of-the-art review. J Am Coll Cardiol. (2020) 75:525–38. doi: 10.1016/j.jacc.2019.11.044, 32029136 PMC7187687

[2] Xu CQ Li J Liang ZQ Zhong YL Zhang ZH Hu XQ . Sirtuins in macrophage immune metabolism: a novel target for cardiovascular disorders. Int J Biol Macromol. (2024) 256:128270. doi: 10.1016/j.ijbiomac.2023.128270, 38000586

[3] Dutt C Nunes Salles JE Joshi S Nair T Chowdhury S Mithal A . Risk factors analysis and management of cardiometabolic-based chronic disease in low- and middle-income countries. Diabetes Metab Syndr Obes. (2022) 15:451–65. doi: 10.2147/DMSO.S333787, 35210795 PMC8858768

[4] Zhong YL Xu CQ Li J Liang ZQ Wang MM Ma C . Mitochondrial dynamics and metabolism in macrophages for cardiovascular disease: a review. Phytomedicine. (2025) 140:156620. doi: 10.1016/j.phymed.2025.156620, 40068296

[5] Gonzalez-Chavez A Chavez-Fernandez JA Elizondo-Argueta S Gonzalez-Tapia A Leon-Pedroza JI Ochoa C. Metabolic syndrome and cardiovascular disease: a health challenge. Arch Med Res. (2018) 49:516–21. doi: 10.1016/j.arcmed.2018.10.003 30528299

[6] Drozdz D Alvarez-Pitti J Wojcik M Borghi C Gabbianelli R Mazur A . Obesity and cardiometabolic risk factors: from childhood to adulthood. Nutrients. (2021) 13:4176. doi: 10.3390/nu13114176, 34836431 PMC8624977

[7] Kalra S Unnikrishnan AG Baruah MP Sahay R Bantwal G. Metabolic and energy imbalance in dysglycemia-based chronic disease. Diabetes Metab Syndr Obes. (2021) 14:165–84. doi: 10.2147/DMSO.S286888, 33488105 PMC7816219

[8] Nieto-Martinez R Gonzalez-Rivas JP Mechanick JI. Cardiometabolic risk: new chronic care models. JPEN J Parenter Enteral Nutr. (2021) 45:85–92. doi: 10.1002/jpen.2264 34519362

[9] Huang X Hu L Tao S Xue T Hou C Li J. Relationship between uric acid to high-density cholesterol ratio (UHR) and circulating α-klotho: evidence from NHANES 2007–2016. Lipids Health Dis. (2024) 23:244. doi: 10.1186/s12944-024-02234-6, 39123222 PMC11312937

[10] Xu L Li C Wan T Sun X Lin X Yan D . Targeting uric acid: a promising intervention against oxidative stress and neuroinflammation in neurodegenerative diseases. Cell Commun Signal. (2025) 23:4. doi: 10.1186/s12964-024-01965-4, 39754256 PMC11699683

[11] Fujii W Yamazaki O Hirohama D Kaseda K Kuribayashi-Okuma E Tsuji M . Gene-environment interaction modifies the association between hyperinsulinemia and serum urate levels through SLC22A12. J Clin Invest. (2025) 135:e186633. doi: 10.1172/JCI186633, 40100301 PMC12077893

[12] Lehti S Korhonen TM Soliymani R Ruhanen H Lahteenmaki E Palviainen M . The lipidome and proteome of high-density lipoprotein are altered in menopause. J Appl Physiol. (2025) 139:308. doi: 10.1152/japplphysiol.00120.2025, 40465474

[13] Mao H Zhang X Huang S Lin T Chen Z. Relationship between uric acid to high-density lipoprotein cholesterol ratio and sarcopenia in NHANES: exploring the mediating role of bilirubin and association with all-cause mortality. Front Nutr. (2025) 12:1560617. doi: 10.3389/fnut.2025.1560617, 40626224 PMC12229869

[14] Wang F Qiao H Zheng Y Zheng Y Ni Y He X. Exploring the nonlinear relationship between serum uric acid to high-density lipoprotein cholesterol ratio and obesity in older adults: a cross-sectional study. Front Public Health. (2025) 13:1587194. doi: 10.3389/fpubh.2025.1587194, 40376050 PMC12078289

[15] Yin J Zheng C Lin X Huang C Hu Z Lin S . The potential of the serum uric acid to high-density lipoprotein cholesterol ratio as a predictive biomarker of diabetes risk: a study based on NHANES 2005-2018. Front Endocrinol (Lausanne). (2024) 15:1499417. doi: 10.3389/fendo.2024.1499417, 39916754 PMC11798810

[16] Li Y Bai L. Association between uric acid to high-density lipoprotein cholesterol ratio and abdominal aortic calcification: a cross-sectional study. J Clin Lipidol. (2025) 19:899–911. doi: 10.1016/j.jacl.2025.05.017, 40579260

[17] Ding L Guo H Zhang C Liang X Liu Y. Association between serum uric acid to high-density lipoprotein cholesterol ratio and all-cause and cardiovascular disease mortality after stroke: a cross-sectional study from 2005 to 2018. Nutr Metab Cardiovasc Dis. (2025) 35:103909. doi: 10.1016/j.numecd.2025.103909, 40087043

[18] Li S Liu Z Lu C. Association of uric acid to high-density lipoprotein cholesterol ratio with the presence or absence of hypertensive kidney function: results from the China health and retirement longitudinal study (CHARLS). BMC Nephrol. (2025) 26:123. doi: 10.1186/s12882-024-03939-7, 40050799 PMC11884189

[19] Liu J Chen K Tang M Mu Q Zhang S Li J . Oxidative stress and inflammation mediate the adverse effects of cadmium exposure on all-cause and cause-specific mortality in patients with diabetes and prediabetes. Cardiovasc Diabetol. (2025) 24:145. doi: 10.1186/s12933-025-02698-5, 40158078 PMC11954339

[20] Sun S Xu H Liu L Luan Z Liu C Zhi F. Association of serum uric acid-to-high-density lipoprotein cholesterol ratio (UHR) with risk of myocardial infarction among individuals with diabetes: a cross-sectional analysis using data from NHANES 2005-2020. Eur J Med Res. (2025) 30:554. doi: 10.1186/s40001-025-02845-4, 40605038 PMC12219884

[21] Zhao P Hu HZ Chen XT Jiang QY Yu XZ Cen XL . Mild hyperthermia enhanced synergistic uric acid degradation and multiple ROS elimination for an effective acute gout therapy. J Nanobiotechnol. (2024) 22:275. doi: 10.1186/s12951-024-02539-9, 38778401 PMC11112921

[22] Pammer A Klobucar I Stadler JT Meissl S Habisch H Madl T . Impaired HDL antioxidant and anti-inflammatory functions are linked to increased mortality in acute heart failure patients. Redox Biol. (2024) 76:103341. doi: 10.1016/j.redox.2024.103341, 39244794 PMC11406013

[23] Yang Y Zhang J Jia L Su J Ma M Lin X. The interaction between uric acid and high-density lipoprotein cholesterol on the prognosis of patients with acute myocardial infarction. Front Cardiovasc Med. (2023) 10:1226108. doi: 10.3389/fcvm.2023.1226108, 37492158 PMC10363914

[24] Roman-Filip I Roman-Filip C Morosanu V Andone S Bajko Z Balasa R. Uric acid in cerebral ischemia: a systematic review of its biomarker value and role in neuroprotection. Int J Mol Sci. (2025) 26:10268. doi: 10.3390/ijms262110268, 41226323 PMC12610115

[25] Schmitt A Behnes M Bertsch T Reinhardt M Goertz M Abel N . Prognostic impact of uric acid levels in heart failure with mildly reduced ejection fraction: insights from a large retrospective registry. Eur J Intern Med. (2025) 140:106404. doi: 10.1016/j.ejim.2025.06.033, 40634144

[26] Rosenson RS Brewer HB Jr Ansell BJ Barter P Chapman MJ Heinecke JW . Dysfunctional HDL and atherosclerotic cardiovascular disease. Nat Rev Cardiol. (2016) 13:48–60. doi: 10.1038/nrcardio.2015.124, 26323267 PMC6245940

[27] He X Zhu J Liang W Yang X Ning W Zhao Z . Association of body roundness index with cardiovascular disease in patients with cardiometabolic syndrome: a cross-sectional study based on NHANES 2009-2018. Front Endocrinol (Lausanne). (2025) 16:1524352. doi: 10.3389/fendo.2025.1524352, 39963283 PMC11830584

[28] Kelebie M Kibralew G Tadesse G Nakie G Ali D Fanta B . Prevalence and predictors of metabolic syndrome among psychiatric patients receiving antipsychotic treatment in Africa: a systematic review and meta-analysis. BMC Psychiatry. (2025) 25:433. doi: 10.1186/s12888-025-06894-1, 40301830 PMC12038947

[29] Ji H Sabanayagam C Matsushita K Cheng CY Rim TH Sheng B . Sex differences in cardiovascular-kidney-metabolic syndrome: 30-year US trends and mortality risks-brief report. Arterioscler Thromb Vasc Biol. (2025) 45:157–61. doi: 10.1161/ATVBAHA.124.321629, 39665141 PMC11729504

[30] Wang Y O'Neil A Jiao Y Wang L Huang J Lan Y . Sex differences in the association between diabetes and risk of cardiovascular disease, cancer, and all-cause and cause-specific mortality: a systematic review and meta-analysis of 5,162,654 participants. BMC Med. (2019) 17:136. doi: 10.1186/s12916-019-1355-0, 31296205 PMC6625042

[31] Wurtz P Cook S Wang Q Tiainen M Tynkkynen T Kangas AJ . Metabolic profiling of alcohol consumption in 9778 young adults. Int J Epidemiol. (2016) 45:1493–506. doi: 10.1093/ije/dyw175, 27494945 PMC5100616

[32] McClements L Kautzky-Willer A Kararigas G Ahmed SB Stallone JN. The role of sex differences in cardiovascular, metabolic, and immune functions in health and disease: a review for "sex differences in health awareness day". Biol Sex Differ. (2025) 16:33. doi: 10.1186/s13293-025-00714-7, 40361226 PMC12076860

[33] Nichols GA Horberg M Koebnick C Young DR Waitzfelder B Sherwood NE . Cardiometabolic risk factors among 1.3 million adults with overweight or obesity, but not diabetes, in 10 geographically diverse regions of the United States, 2012-2013. Prev Chronic Dis. (2017) 14:E22. doi: 10.5888/pcd14.160438 28278130 PMC5345964

[34] Xia TL Li YM Huang FY Chai H Huang BT Li Q . The triglyceride paradox in the mortality of coronary artery disease. Lipids Health Dis. (2019) 18:21. doi: 10.1186/s12944-019-0972-0, 30670053 PMC6343235

[35] Li H Jiang Y Su X Meng Z. The triglyceride glucose index was U-shape associated with all-cause mortality in population with cardiovascular diseases. Diabetol Metab Syndr. (2023) 15:181. doi: 10.1186/s13098-023-01153-3, 37679825 PMC10483863

[36] Li S Zhang Y Luo D Lai C Chen B. Correlation between serum uric acid to high-density lipoprotein cholesterol ratio and cardiometabolic multimorbidity in China: a nationwide longitudinal cohort study. Nutr Metab Cardiovasc Dis. (2025) 35:103865. doi: 10.1016/j.numecd.2025.103865, 39988508

[37] Du Y Wang K. Machine learning prediction of chronic kidney disease in elderly MetS patients using NHANES 2011-2020 data. Rejuvenation Res. (2025). doi: 10.1177/15491684251399607, 41371294

[38] Nakhleh A Sakhnini R Furman E Shehadeh N. Cardiometabolic risk factors among children and adolescents with overweight and class 1 obesity: a cross-sectional study. Insights from stratification of class 1 obesity. Front Endocrinol (Lausanne). (2023) 14:1108618. doi: 10.3389/fendo.2023.1108618 36798669 PMC9927000

